# Editorial: New diagnostic perspectives in urogenital radiology

**DOI:** 10.3389/fmed.2023.1280300

**Published:** 2023-11-08

**Authors:** Mohamed Shehata, Rasha T. Abouelkheir, Mostafa Elhosseini

**Affiliations:** ^1^Department of Bioengineering, Speed School of Engineering, University of Louisville, Louisville, KY, United States; ^2^Department of Computers and Control Systems Engineering, Faculty of Engineering, Mansoura University, Mansoura, Egypt; ^3^Department of Radiology, Urology and Nephrology Center, Mansoura University, Mansoura, Egypt; ^4^College of Computer Science and Engineering, Taibah University, Yanbu, Saudi Arabia

**Keywords:** artificial intelligence, computer-aided detection, renal cancer, deep learning, MRI, urogenital abnormalities, prostate cancer, COVID-19

## 1. Introduction

Urogenital Radiology, a key area of medical imaging, focuses on diagnosing and treating urinary and reproductive system conditions. Its relevance has surged due to the rise in global urogenital disorders. The field has evolved with the adoption of advanced diagnostic tools like ultrasound, CT scans, and MRI. Ultrasound offers non-invasive, real-time imaging, while CT scans and MRI provide detailed images, aiding in the diagnosis of conditions like kidney stones, tumors, and infections. Historically, urogenital radiology began with Wilhelm Röntgen's discovery of X-rays in 1895, transforming medical diagnostics. While X-rays remain vital, the 1970s saw the introduction of CT scans and MRI, enhancing image quality and clarity. However, the early years posed challenges, such as subpar image quality and radiation risks from X-rays, especially for children. The evolution of imaging, marked by CT and MRI advancements and new contrast agents, has significantly improved diagnostic accuracy in urogenital radiology. These innovations promise further refinement, ensuring timely diagnoses and better patient outcomes.

## 2. Overview of the contributing articles

Researchers at King Abdullah University Hospital (KAUH) conducted a retrospective study comparing imaging volumes during the peak of the COVID-19 pandemic in 2020 to the same period in 2019 (Gharaibeh et al.). Findings revealed a 29.4% reduction in 2020, with nuclear imaging seeing the most significant drop at 41.0%. This study highlights the considerable impact of the pandemic on radiology services in Jordan, emphasizing the need for strategies to maintain healthcare services during future crises. The data is crucial for healthcare decision-makers to ensure continued care and timely interventions during similar events.

The study introduced an AI-based transfer learning framework for early renal disease detection using CT scans and histopathological images (Badawy et al.). Leveraging convolutional neural networks (CNN) and optimization algorithms, it achieved near-perfect accuracy rates of 99.98% and 100% for different datasets. This research highlights the growing significance of AI in healthcare, with the potential to set new standards in renal disease diagnosis due to its high accuracy and adaptability. The study's findings emphasize the future of personalized and efficient care in the realm of renal diseases.

The study utilized region-specific U-Net deep learning models to segment rectal structures on post-treatment T2-weighted MRI scans (DeSilvio et al.). These models demonstrated high accuracy, outperforming traditional methods and offering consistent results across different imaging settings. This advancement in precision oncology can improve treatment plans and patient outcomes for rectal cancers, emphasizing the potential of deep learning in medical imaging.

The study compared the diagnostic performance of 18F-DCFPyL PET/CT and 99mTc-MDP SPECT/CT bone imaging in detecting bone metastases in prostate cancer patients (Hu et al.). 18F-DCFPyL PET/CT showed superior accuracy in both patient and lesion levels. This suggests that 18F-DCFPyL PET/CT could be the preferred imaging choice for early detection, leading to tailored treatments and better patient outcomes in prostate cancer.

## 3. Emerging trends and technologies

Urogenital radiology is undergoing significant technological advancements. The integration of PET/CT has enhanced cancer imaging, especially in prostate cancer studies (Hu et al.). Magnetic Resonance Urography (MRU) provides exceptional clarity for the urinary tract, especially for structures like the kidneys and bladder (Gharaibeh et al.). The advent of 3D and 4D imaging offers spatial and real-time visualization, aiding complex diagnoses and treatments (DeSilvio et al.). Intraoperative imaging has improved surgical precision, while AI and Machine Learning are reshaping urogenital radiology, with research highlighting their role in refining diagnostics and treatments (Badawy et al.). The future in this field is bright, with studies emphasizing the superiority of techniques like 18F-DCFPyL PET/CT (Hu et al.), the potential of AI-driven systems in kidney stone treatments (Badawy et al.), and the combined benefits of deep learning and advanced imaging in urological procedures (DeSilvio et al.). Real-time surgical imaging further promises enhanced patient outcomes.

## 4. Challenges and future directions

Urogenital radiology is at a pivotal juncture, offering both promising opportunities and challenges. New imaging techniques, while advanced, are costly, prompting exploration of solutions like modular upgrades and group purchasing. A global disparity in accessing these techniques exists, emphasizing the need for stronger public-private and international collaborations. As new methods emerge, continuous training for radiologists becomes crucial, and patient apprehensions about these techniques highlight the need for better education and communication. Integrating AI into radiology demands collaboration among radiologists, AI specialists, and administrators, and ongoing evaluation of imaging techniques is essential. Research is focusing on refining AI for radiology, assessing transfer learning, and expanding region-specific deep learning in urogenital imaging. The importance of flexible radiology models, especially post-COVID-19, AI-integrated apps, and broader equipment distribution is evident. However, gaps remain, including the need to explore AI's broader role, standardize deep learning models, compare new and traditional techniques, and address research biases while expanding study cohorts.

## 5. Conclusion

Urogenital radiology has evolved from basic X-rays to advanced techniques like MRI and CT, enhancing diagnostic precision. Recent research highlights innovations like the DRDC system for renal diseases and deep learning for rectal imaging. While emerging trends like PET/CT promise further advancements, challenges in cost and accessibility persist. Addressing these requires a focus on affordability and patient education. Collaborative efforts are essential to navigate uncertainties and maximize the benefits of these innovations.

## Author contributions

MS: Conceptualization, Project administration, Writing—original draft, Writing—review & editing, Investigation, Supervision. RA: Conceptualization, Project administration, Writing—original draft, Writing—review & editing, Investigation, Supervision. ME: Conceptualization, Investigation, Project administration, Supervision, Writing—original draft, Writing—review & editing.

